# Expression of IL4Rα and IL13Rα1 are associated with poor prognosis of soft-tissue sarcoma of the extremities, superficial trunk, and retroperitoneum

**DOI:** 10.1186/s13000-020-01066-z

**Published:** 2021-01-09

**Authors:** Kyoung Min Kim, Usama Khamis Hussein, See-Hyoung Park, Young Jae Moon, Zhongkai Zhang, Asmaa Gamal Ahmed, Ae-Ri Ahn, Ho Sung Park, Jung Ryul Kim, Kyu Yun Jang

**Affiliations:** 1 Department of Pathology, Jeonbuk National University Medical School, 567 Baekje-daero, Dukjin-gu, Jeonju, 54896 Republic of Korea; 2 Research Institute of Clinical Medicine of Jeonbuk National University-Biomedical, Research Institute of Jeonbuk National University Hospital and Research Institute for Endocrine Sciences, Jeonju, Republic of Korea; 3 Faculty of Science, Beni-Suef University, Beni-Suef, Egypt; 4 Department of Bio and Chemical Engineering, Hongik University, Sejong, Republic of Korea; 5 Department of Biochemistry and Molecular Biology, Jeonbuk National University Medical School, Jeonju, Republic of Korea; 6 Department of Orthopedic Surgery, Jeonbuk National University Medical School, 567 Baekje-daero, Dukjin-gu, Jeonju, 54896 Republic of Korea; 7 Faculty of Postgraduate Studies and Advanced Sciences, Beni-Suef University, Beni-Suef, Egypt

**Keywords:** Soft tissue, Sarcoma, IL4Rα, IL13Rα1, Prognosis

## Abstract

**Background:**

IL4Rα and IL13Rα1 are constituents of the type II IL4 receptor. Recently, IL4Rα and IL13Rα1 were reported to have roles in cancer progression and suggested as potential prognostic markers. However, studies on IL4Rα and IL13Rα1 in soft-tissue sarcomas have been limited.

**Methods:**

This study investigated the immunohistochemical expression of IL4Rα and IL13Rα1 in 89 soft-tissue sarcomas of the extremities, superficial trunk, and retroperitoneum. Immunohistochemical staining for IL4Rα and IL13Rα1 were scored according to a combination of staining intensity and staining area in tissue microarray samples. Positivity for the immunohistochemical expression of IL4Rα and IL13Rα1 were determined using receiver operating curve analysis. Statistical analysis was performed using regression analysis and a chi-square test.

**Results:**

In human soft-tissue sarcomas, immunohistochemical expression of IL4Rα was significantly associated with IL13Rα1 expression. Nuclear and cytoplasmic expression of IL4Rα and IL13Rα1 were significantly associated with shorter survival of soft-tissue sarcoma patients in univariate analysis. Multivariate analysis indicated that nuclear expression of IL4Rα and IL13Rα1 were independent indicators of shorter overall survival (IL4Rα; *p* = 0.002, IL13Rα1; *p* = 0.016) and relapse-free survival (IL4Rα; *p* = 0.022, IL13Rα1; *p* < 0.001) of soft-tissue sarcoma patients. Moreover, the co-expression pattern of nuclear IL4Rα and IL13Rα1 was an independent indicator of shorter survival of soft-tissue sarcoma patients (overall survival; overall *p* < 0.001, relapse-free survival; overall *p* < 0.001).

**Conclusions:**

This study suggests IL4Rα and IL13Rα1 are associated with the progression of soft-tissue sarcoma, and the expression of IL4Rα and IL13Rα1 might be novel prognostic indicators of soft-tissue sarcoma patients.

## Background

Cytokines and cytokine receptors have crucial roles in the regulation of the biologic mechanisms of immune cells and tumor cells [1, 2]. Recent advances in cancer biology reveal that cytokines and their receptors mediate cancer-related signaling. Especially, the IL4 receptor (IL4R) complex has been studied for its role in cancer progression [2, 3]. There are three types of receptor complexes that IL4 binds to [3, 4]. Type I IL4R is expressed on T-cells and NK cells and is composed of IL4Rα and IL2Rγc [1, 3, 4]. Type II IL4R is expressed on solid tumors and fibroblasts and is composed of IL4Rα and IL13Rα1 [2, 4, 5]. Type III IL4R is expressed on B-cells and monocytes and is composed of IL4Rα, IL13Rα1, and IL2Rγc [3]. Among these three types of IL4R complexes, type II IL4R is activated by binding of both IL4 and IL13, and studies on type II IL4R have focused on its expression on solid tumors [1–4, 6]. Higher expression of IL4Rα and IL13Rα1 was observed in various types of human cancers such as colorectal, breast, pancreatic, bladder, brain, and ovarian cancers [3, 7, 8]. In addition, elevated expression of IL4Rα and/or IL13Rα1 were associated with poor prognosis of glioblastoma [9], mesothelioma [10], breast cancer [11], renal cell carcinoma [12], and oral cavity squamous cell carcinoma patients [13]. These clinical impact of the IL4Rα and IL13Rα1 expression in human cancer has been associated with the role of IL4Rα/IL13Rα1 receptor complex in the proliferation and survival of cancer cells. The activation of IL4Rα/IL13Rα1 by IL4/IL13 stimulates JAK1/JAK2/JAK3-STAT6-mediated proliferation of cancer cells [1–3, 14]. Therefore, based on the relationship between the IL4/IL13, IL4R receptor complex, and JAK1/JAK2/JAK3-STAT6, there have been multiple clinical trials to treat human cancers via inhibition or blocking of the IL4Rα/IL13Rα1 pathway [1–3].

Soft-tissue sarcoma (STS) is a malignant tumor that originates from various types of mesenchymal tissue. Therefore, STS is not a single type of malignant tumor, but includes multiple types of soft tissue malignant tumors with diverse backgrounds [15]. However, despite the heterogeneity of this tumor type and the numerous histologic subtypes of STS, the incidence of STS is very low [16]. In addition, the study of the pathogenesis of STSs to achieve successful treatment of STSs is limited. Therefore, further study on the treatment of STS is needed. There have been recent advances in the understating of the role of cytokines and cytokine receptors in the development and progression of human cancers. Among the mesenchymal type of cancers such as rhabdomyosarcoma and osteosarcoma cells, it has been suggested that the IL4R pathway might be a therapeutic target of these types of cancers [16, 17]. Therefore, based on the structural relationship of IL4Rα and IL13Rα1 as the components of type II IL4R [1–3], we investigated the expression and clinicopathological significance of IL4Rα and IL13Rα1 in human STSs.

## Methods

### Soft-tissue sarcoma patients

In this study, STSs diagnosed and treated between July 1998 and January 2013 at Jeonbuk National University Hospital were evaluated. STSs were reviewed according to the latest WHO classification of soft tissue tumors [15] and the 8th edition of the American Joint Committee Cancer Staging System [18]. Thereafter, specific subtypes of STS such as a gastrointestinal stromal tumor, Kaposi sarcoma, and atypical lipomatous tumor were not included in this study. Gastrointestinal stromal tumors are treated with targeted therapeutics, and the classification of risk category of the gastrointestinal stromal tumor differs from the staging of conventional STSs [15, 18]. Kaposi sarcoma is uniformly associated with HHV8 infection, and atypical lipomatous tumor is not classified as a malignant tumor in the latest WHO classification [15]. In addition, because there are no stages for STSs of the head and neck, thoracic, and abdominal viscera in the latest staging system, the STSs of the extremities, superficial trunk, and retroperitoneum were included in this study [15, 18]. Finally, 89 cases of STSs with a complete medical history, histologic slides, and tissue blocks were included in this study. Information regarding clinicopathological variables of the STSs was obtained by review of the medical records. The clinicopathological factors considered in this study were the age of the patients, sex, tumor stage, tumor size, lymph node metastasis at diagnosis, distant metastasis at diagnosis, histologic grade, tumor differentiation, mitotic count, tumor necrosis, and histologic subtype of STS. The age of the STS patients included in this study ranged from 2 months to 84 years (mean age; 50.6 years, median age; 53.0 years). This study was performed with approval by the institutional review board of Jeonbuk National University Hospital (IRB number, CUH 2015–09–024-002) and was performed in compliance with the Declaration of Helsinki. In this approval, written informed consent was waived because of the anonymous and retrospective nature of this study.

### Immunohistochemical staining and scoring

The expression of IL4Rα and IL13Rα1 in STSs was evaluated by immunohistochemical staining of tissue microarray (TMA) sections. The TMA cores were obtained from the original paraffin-embedded tissue block after a review of original histologic slides. Two 3.0 mm cores per case were used to establish a TMA block from the area with the highest histologic grade without any degenerative change. The TMA tissue sections were deparaffinized and underwent an antigen retrieval procedure by boiling for 20 min with a microwave oven in a pH 6.0 antigen retrieval solution (DAKO, Glostrup, Denmark). The tissue sections were incubated with primary antibodies for IL4Rα (1:100, sc-165,974, Santa Cruz Biotechnology, Santa Cruz, CA) and IL13Rα1 (1:100, sc-25,849, Santa Cruz Biotechnology, Santa Cruz, CA) and visualized with the DAKO Envision system (DAKO, Carpinteria, CA). Immune-stained slides were scored according to staining intensity and stained area according to their expression in the cytoplasm or nuclei of tumor cells. The staining intensity was scored from zero to three (0; no staining, 1; weak staining, 2; intermediate staining, 3; strong staining) and the stained area was scored from zero to five (0; 0%, 1; 1%, 2; 2–10%, 3; 11–33%, 4; 34–66%, 5; 67–100%) [19–22]. The immunohistochemical staining score in each TMA core was obtained by adding a staining intensity score and the stained area score. Thereafter, because we used two TMA cores in each case, the sum of the immunohistochemical staining scores from two TMA cores was used as final immunohistochemical score. The scoring of the immunohistochemically stained slides was performed by two pathologists (KYJ and KMK) by simultaneous observation under a multi-viewing microscope without knowledge of the clinicopathological information. The scores were obtained with a consensus of two pathologists.

### Statistical analysis

The positivity of immunohistochemical expression of IL4Rα and IL13Rα1 was determined by using receiver operating characteristic (ROC) curve analysis [23–25]. An event in ROC curve analysis was defined as the death of a patient by STS, and the cut-off point was determined at the point with the highest area under the curve (AUC) [23, 25]. The end-point of follow up was June 2014. The prognosis of STS patients was evaluated for overall survival (OS) and relapse-free survival (RFS). An event in OS analysis was the death of a patient from STS. The patients who were alive at the end-point of follow-up or died by other causes were censored. The duration of follow-up for OS analysis was determined from the date of operation to the date of the last contact. An event in RFS was a relapse of STS or death of the patients from STS. The patients who were alive without relapse at the end-point of follow-up or died by other causes were censored. The duration of follow-up for RFS analysis was determined from the date of operation to the date of the event or last contact. The prognostic values of potential prognostic factors were evaluated by performing univariate and multivariate Cox proportional hazards regression analysis and Kaplan-Meier survival analysis. The relationships between the potential prognostic clinicopathological factors were determined via Pearson’s chi-square test. Statistical analysis was performed with SPSS software (IBM, version 22.0, Armonk, NY) and *p* values less than 0.05 being considered statistically significant.

## Results

### The association between the clinicopathologic variables and the expression of IL4Rα and IL13Rα1 in soft-tissue sarcomas

**Fig. 1:**
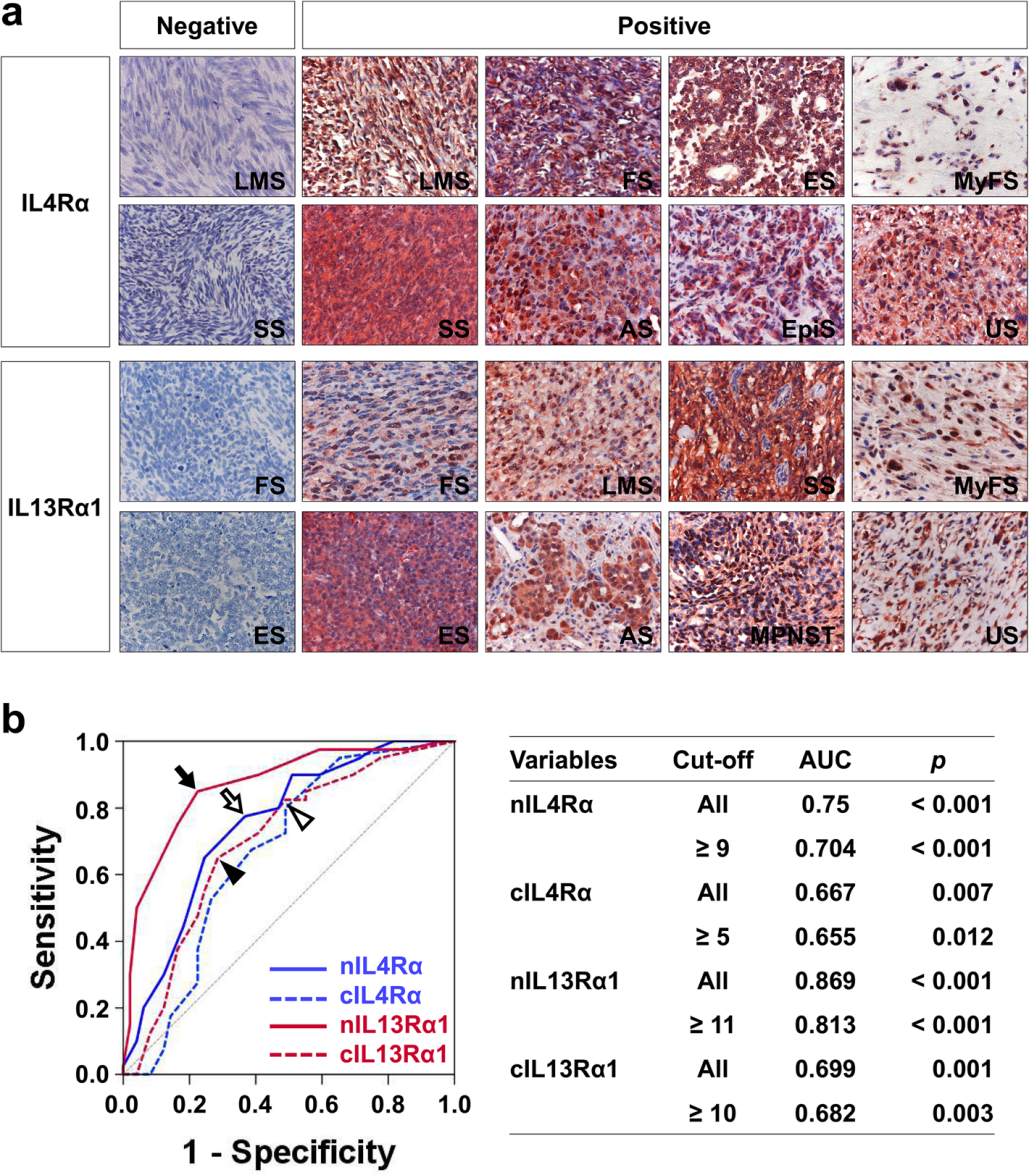
Immunohistochemical expression of IL4Rα and IL13Rα1 in soft-tissue sarcomas and statistical analysis. **a** Immunohistochemical expression of IL4Rα and IL13Rα1 in various histologic types of soft-tissue sarcomas. IL4Rα and IL13Rα1are expressed in both the cytoplasm and nuclei of tumor cells. Original magnification: × 400. **b** Receiver operating characteristic curve analysis to determine cut-off points for the expression of nuclear IL4Rα (nIL4Rα, empty arrow), cytoplasmic IL4Rα (cIL4Rα, empty arrowhead), nuclear IL13Rα1 (nIL13Rα1, black arrow), and cytoplasmic IL13Rα1 (cIL13Rα1, black arrowhead). The cut-off points indicate the point of highest area under the curve (AUC) to predict the death of soft-tissue sarcoma patients. AS; angiosarcoma, ES; Ewing sarcoma, EpiS; Epithelioid sarcoma, FS; fibrosarcoma, LMS; leiomyosarcoma, MPNST; malignant peripheral nerve sheath tumor, MyFS; myxofibrosarcoma, SS; synovial sarcoma, US; undifferentiated sarcoma

The immunohistochemical expression of IL4Rα and IL13Rα1 was seen in both the cytoplasm and the nuclei of tumor cells and representative images for the expression of IL4Rα and IL13Rα1 are presented in Fig. 1a. The cut-off points for the nuclear expression of IL4Rα (nIL4Rα), cytoplasmic expression of IL4Rα (cIL4Rα), nuclear expression of IL13Rα1 (nIL13Rα1), and cytoplasmic expression of IL13Rα1 (cIL13Rα1) were determined by ROC curve analysis (Fig. 1b). The cut-off points for nIL4Rα, cIL4Rα, nIL13Rα1, and cIL13Rα1 were nine, five, eleven, and ten, respectively (Fig. 1b). With these cut-off values, the positivity for nIL4Rα, cIL4Rα, nIL13Rα1, and cIL13Rα1 in various histologic subtypes of STS are presented in Table 1. Positivity for nIL4Rα was significantly associated with age (*p =* 0.033), higher tumor stage (*p <* 0.001), lymph node metastasis (*p =* 0.034), higher histologic grade (*p =* 0.002), increased mitotic count (*p <* 0.001), presence of tumor necrosis (*p =* 0.020), and expression of cIL4Rα (*p <* 0.001), nIL13Rα1 (*p <* 0.001), and cIL13Rα1 (*p =* 0.003) (Table 2). Positivity for cIL4Rα was significantly associated with higher tumor stage (*p =* 0.012), lymph node metastasis (*p =* 0.027), higher histologic grade (*p <* 0.001), increased mitotic count (*p =* 0.003), presence of tumor necrosis (*p =* 0.022), and expression of nIL13Rα1 (*p <* 0.001) and cIL13Rα1 (*p <* 0.001) (Table 2). The expression of nIL13Rα1 was significantly associated with higher tumor stage (*p <* 0.001), lymph node metastasis (*p =* 0.015), distant metastasis (*p <* 0.001), higher histologic grade (*p <* 0.001), increased mitotic count (*p =* 0.002), presence of tumor necrosis (*p <* 0.001), and expression of cIL13Rα1 (*p <* 0.001) (Table 2). The expression of cIL13Rα1 was significantly associated with higher histologic grade (*p =* 0.030) (Table 2).

**Table 1:** The expression of IL4Rα and IL13Rα1 according to the histologic subtype of soft-tissue sarcomas

Histologic type	No.	nIL4Rα		cIL4Rα		nIL13Rα1		cIL13Rα1	
		positive	*p*	positive	*p*	positive	*p*	positive	*p*
Synovial sarcoma	15	10 (67%)	0.027	12 (80%)	< 0.001	8 (53%)	0.010	8 (53%)	0.077
Leiomyosarcoma	13	8 (62%)		8 (62%)		10 (77%)		8 (62%)	
Undifferentiated sarcoma	10	8 (80%)		9 (90%)		7 (70%)		7 (70%)	
Liposarcoma, myxoid	9	0 (0%)		0 (0%)		0 (0%)		1 (11%)	
Liposarcoma, WD	4	1 (25%)		1 (25%)		1 (25%)		0 (0%)	
Liposarcoma, dedifferentiated	3	0 (0%)		0 (0%)		1 (33%)		1 (33%)	
Rhabdomyosarcoma, alveolar	3	1 (33%)		3 (100%)		1 (33%)		1 (33%)	
Rhabdomyosarcoma, embryonal	2	1 (50%)		2 (100%)		0 (0%)		0 (0%)	
Rhabdomyosarcoma, pleomorphic	1	0 (0%)		1 (100%)		0 (0%)		0 (0%)	
Rhabdomyosarcoma, spindle	1	1 (100%)		1 (100%)		1 (100%)		1 (100%)	
Myxofibrosarcoma	6	4 (67%)		3 (50%)		2 (33%)		2 (33%)	
MPNST	5	2 (40%)		1 (20%)		3 (60%)		1 (20%)	
Epithelioid sarcoma	4	3 (75%)		3 (75%)		4 (100%)		4 (100%)	
Ewing sarcoma	4	3 (75%)		3 (75%)		3 (75%)		2 (50%)	
Fibrosarcoma^a^	4	2 (50%)		4 (100%)		1 (25%)		2 (50%)	
Angiosarcoma	3	3 (100%)		3 (100%)		3 (100%)		2 (67%)	
Low-grade myofibroblastic sarcoma	2	2 (100%)		2 (100%)		0 (0%)		0 (0%)	

Abbreviations: *WD* Well differentiated, *MPNST* Malignant peripheral nerve sheath tumor, *nIL4Rα* Nuclear expression of IL4Rα, *cIL4Rα* Cytoplasmic expression of IL4Rα, *nIL13Rα1* Nuclear expression of IL13Rα1, *cIL13Rα1* Cytoplasmic expression of IL13Rα1. ^a^The four cases of fibrosarcoma consist of one case of infantile fibrosarcoma and three cases of adult fibrosarcoma

**Table 2:** Clinicopathologic variables and the expression of IL4Rα and IL13Rα1 in soft-tissue sarcomas

Characteristics		No.	nIL4Rα		cIL4Rα		nIL13Rα1		cIL13Rα1	
			positive	*p*	positive	*p*	positive	*p*	positive	*p*
Age, y	< 60	56	26 (46%)	0.033	33 (59%)	0.310	26 (46%)	0.310	22 (39%)	0.162
	≥ 60	33	23 (70%)		23 (70%)		19 (58%)		18 (55%)	
Sex	Female	37	19 (51%)	0.553	22 (59%)	0.568	15 (41%)	0.111	13 (35%)	0.117
	Male	52	30 (58%)		34 (65%)		30 (58%)		27 (52%)	
Stage	I and II	36	12 (33%)	< 0.001	17 (47%)	0.012	8 (22%)	< 0.001	12 (33%)	0.070
	III and IV	53	37 (70%)		39 (74%)		37 (70%)		28 (53%)	
Tumor size	≤ 5 cm	35	15 (43%)	0.063	20 (57%)	0.364	14 (40%)	0.109	16 (46%)	0.906
	> 5 cm	54	34 (63%)		36 (67%)		31 (57%)		24 (44%)	
LN metastasis	Absence	77	39 (51%)	0.034	45 (58%)	0.027	35 (45%)	0.015	34 (44%)	0.705
	Presence	12	10 (83%)		11 (92%)		10 (83%)		6 (50%)	
Distant metastasis	Absence	65	32 (49%)	0.069	38 (58%)	0.152	23 (35%)	< 0.001	26 (40%)	0.123
	Presence	24	17 (71%)		18 (75%)		22 (92%)		14 (58%)	
Histological grade	Low	18	4 (22%)	0.002	5 (28%)	< 0.001	2 (11%)	< 0.001	4 (22%)	0.030
	High	71	45 (63%)		51 (72%)		43 (61%)		36 (51%)	
Tumor differentiation	1	7	4 (57%)	0.908	4 (57%)	0.742	2 (29%)	0.742	1 (14%)	0.225
	2 and 3	82	45 (55%)		52 (63%)		52 (63%)		43 (52%)	
Mitotic count	0–9/10 HPF	36	12 (33%)	< 0.001	16 (44%)	0.003	11 (31%)	0.002	13 (36%)	0.167
	> 9/10 HPF	53	37 (70%)		40 (75%)		34 (64%)		27 (51%)	
Tumor necrosis	Absence	48	21 (44%)	0.020	25 (52%)	0.022	16 (33%)	< 0.001	17 (35%)	0.051
	Presence	41	28 (68%)		31 (76%)		29 (71%)		23 (56%)	
cIL13Rα1	Negative	49	20 (41%)	0.003	23 (47%)	< 0.001	14 (29%)			
	Positive	40	29 (73%)		33 (83%)		31 (78%)	< 0.001		
nIL13Rα1	Negative	44	13 (30%)	< 0.001	18 (41%)	< 0.001				
	Positive	45	36 (80%)		38 (84%)					
cIL4Rα	Negative	33	3 (9%)	< 0.001						
	Positive	56	46 (82%)							

Abbreviations: *nIL4Rα* Nuclear expression of IL4Rα, *cIL4Rα* Cytoplasmic expression of IL4Rα, *nIL13Rα1* nuclear expression of IL13Rα1, *cIL13Rα1* Cytoplasmic expression of IL13Rα1

### The expressions of IL4Rα and IL13Rα1 are associated with shorter survival of soft-tissue sarcoma patients

**Fig. 2:**
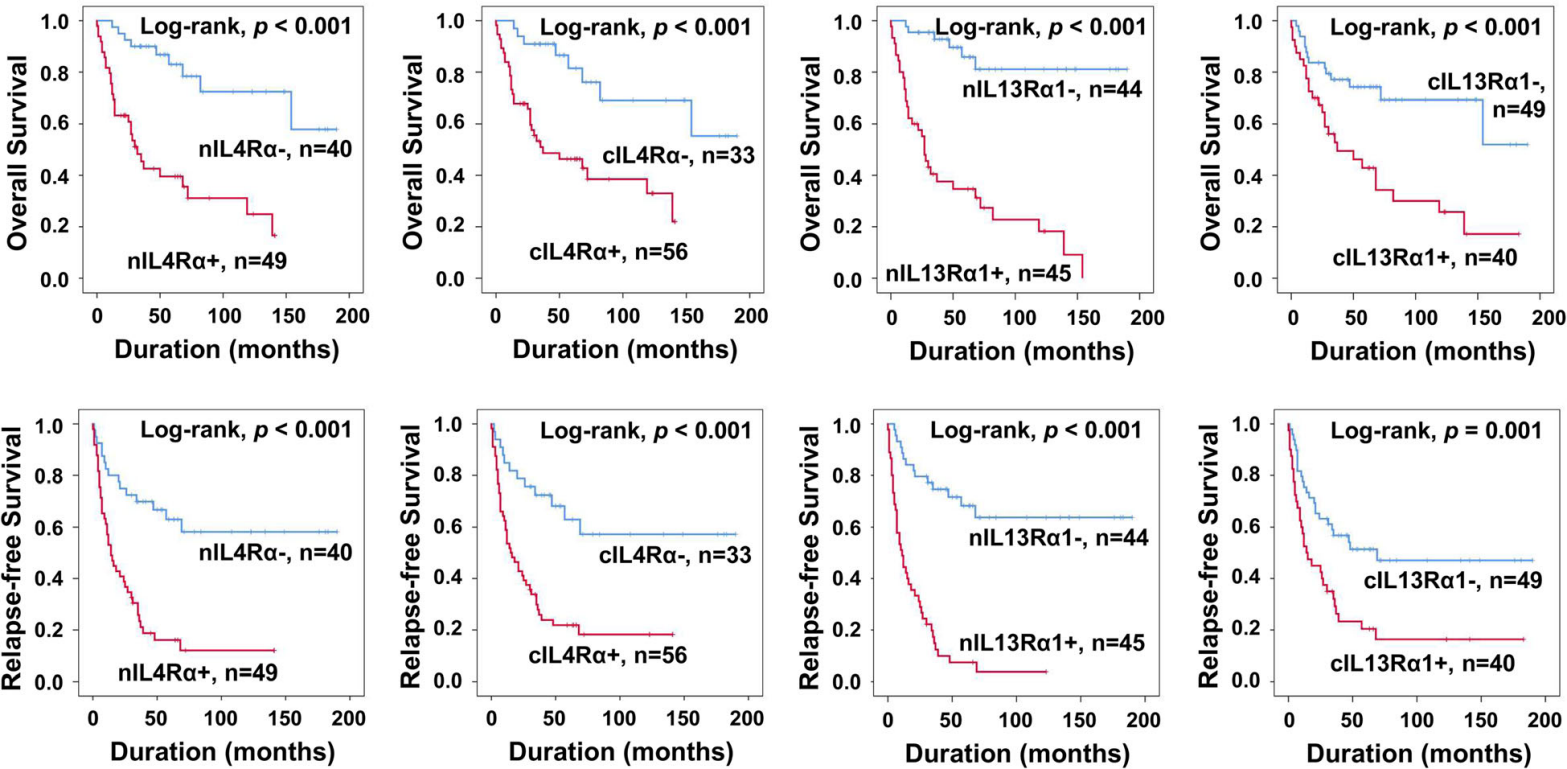
Survival analysis of overall survival and relapse-free survival in soft tissue sarcoma patients according to the expression of IL4Rα and IL13Rα1. Kaplan-Meier survival curves according to the expression of nuclear IL4Rα (nIL4Rα), cytoplasmic IL4Rα (cIL4Rα), nuclear IL13Rα1 (nIL13Rα1), and cytoplasmic IL13Rα1 (cIL13Rα1)

The factors significantly associated with OS or RFS in univariate analysis were age (OS; *p =* 0.379, RFS; *p =* 0.047), tumor stage (OS; *p <* 0.001, RFS; *p =* 0.004), lymph node metastasis (OS; *p =* 0.006, RFS; *p =* 0.038), distant metastasis (OS; *p <* 0.001, RFS; *p <* 0.001), histologic grade (OS; *p =* 0.006, RFS; *p =* 0.006), mitotic count (OS; *p =* 0.002, RFS; *p =* 0.008), tumor necrosis (OS; *p <* 0.001, RFS; *p =* 0.004), and the expression of nIL4Rα (OS; *p <* 0.001, RFS; *P* < 0.001), cIL4Rα (OS; *p <* 0.001, RFS; *p <* 0.001), nIL13Rα1 (OS; *p <* 0.001, RFS; *P* < 0.001), and cIL13Rα1 (OS; *p =* 0.001, RFS; *p =* 0.002) (Table 3). The nIL4Rα-positivity predicted a 5.249-fold [95% CI (95% confidential interval); 2.398–11.493] greater risk of death and a 3.750-fold (95% CI; 2.051–6.855) greater risk of relapse or death of STS patients (Table 3). The cIL4Rα-positivity predicted a 4.099-fold (95% CI; 1.799–9.339) greater risk of death and a 3.394-fold (95% CI; 1.782–6.464) greater risk of relapse or death of STS patients (Table 3). The nIL13Rα1-positivity predicted a 9.451-fold (95% CI; 3.938–22.683) greater risk of death and a 6.546-fold (95% CI; 3.499–12.248) greater risk of relapse or death of STS patients (Table 3). The cIL13Rα1-positivity predicted a 2.902-fold (95% CI; 1.510–5.579) greater risk of death and a 2.305-fold (95% CI; 1.353–3.924) greater risk of relapse or death of STS patients (Table 3). The Kaplan-Meier survival curves according to the expression of nIL4Rα, cIL4Rα, nIL13Rα1, and cIL13Rα1 are presented in Fig. 2.

**Table 3:** Univariate Cox proportional hazards regression analysis of overall survival and relapse-free survival in soft-tissue sarcoma patients

Characteristics	No.	OS		RFS	
		HR (95% CI)	*p*	HR (95% CI)	*p*
Sex, male (vs. female)	52/89	1.598 (0.834–3.064)	0.158	1.239 (0.724–2.118)	0.435
Age, ≥ 60 (vs. < 60)	23/89	1.330 (0.705–2.512)	0.379	1.714 (1.007–2.917)	0.047
Stage, III and IV (vs. I and II)	53/89	4.540 (1.986–10.375)	< 0.001	2.342 (1.303–4.208)	0.004
Tumor size, > 5 cm (vs. ≤ 5 cm)	54/89	1.582 (0.802–3.120)	0.186	1.157 (0.672–1.991)	0.600
LN metastasis, presence (vs. absence)	12/89	2.834 (1.340–5.994)	0.006	2.076 (1.043–4.134)	0.038
Distant metastasis, presence (vs. absence)	24/89	5.976 (3.152–11.331)	< 0.001	4.097 (2.351–7.139)	< 0.001
Histological grade, high (vs. low)	71/89	16.434 (2.237–120.748)	0.006	3.313 (1.411–7.777)	0.006
Tumor differentiation, 2 and 3 (vs. 1)	82/89	2.041 (0.491–8.481)	0.326	0.899 (0.357–2.260)	0.820
Mitotic count, ≥ 10/10 HPF (vs. 0–9/10 HPF)	53/89	3.548 (1.620–7.772)	0.002	2.196 (1.225–3.937)	0.008
Tumor necrosis, presence (vs. absence)	41/89	4.792 (2.331–9.853)	< 0.001	2.209 (1.294–3.771)	0.004
nIL4Rα, positive (vs. negative)	49/89	5.249 (2.398–11.493)	< 0.001	3.750 (2.051–6.855)	< 0.001
cIL4Rα, positive (vs. negative)	56/89	4.099 (1.799–9.339)	< 0.001	3.394 (1.782–6.464)	< 0.001
nIL13Rα1, positive (vs. negative)	45/89	9.451 (3.938–22.683)	< 0.001	6.546 (3.499–12.248)	< 0.001
cIL13Rα1, positive (vs. negative)	40/89	2.902 (1.510–5.579)	0.001	2.305 (1.353–3.924)	0.002

Abbreviations: *OS* Overall survival, *RFS* Relapse-free survival, *HR* Hazard ratio, *95% CI* 95% confidence interval, *nIL4Rα* Nuclear expression of IL4Rα, *cIL4Rα* Cytoplasmic expression of IL4Rα, *nIL13Rα1* Nuclear expression of IL13Rα1, *cIL13Rα1* Cytoplasmic expression of IL13Rα1

Multivariate analysis was performed with the factors significantly associated with OS or RFS, which were age, tumor stage, lymph node metastasis, distant metastasis, histologic grade, tumor necrosis, and the expression of nIL4Rα, cIL4Rα, nIL13Rα1, and cIL13Rα1. Multivariate analysis revealed distant metastasis, nIL4Rα expression, and nIL13Rα1 expression as independent prognostic indicators of OS and RFS of STS patients (Table 4). The STS patients with nIL4Rα-positive tumors had a 3.920-fold (*p =* 0.002, 95% CI; 1.676–9.167) greater risk in OS analysis and a 2.196-fold (*p =* 0.022, 95% CI; 1.119–4.308) greater risk in RFS analysis compared with nIL4Rα-negative STS patients (Table 4). The STS patients with nIL13Rα1-positive tumor had a 3.397-fold (*p =* 0.016, 95% CI; 1.259–9.164) greater risk in OS analysis and a 3.554-fold (*p <* 0.001, 95% CI; 1.695–7.451) greater risk in RFS analysis compared with nL13Rα1-negative STS patients (Table 4).

**Table 4:** Multivariate Cox regression analysis of overall survival and relapse-free survival in soft-tissue sarcoma patients

Characteristics	OS		RFS	
	HR (95% CI)	*p*	HR (95% CI)	*p*
Distant metastasis, presence (vs. absence)	3.665 (1.747–7.689)	< 0.001	2.160 (1.178–3.958)	0.013
nIL4Rα, positive (vs. negative)	3.920 (1.676–9.167)	0.002	2.196 (1.119–4.308)	0.022
nIL13Rα1, positive (vs. negative)	3.397 (1.259–9.164)	0.016	3.554 (1.695–7.451)	< 0.001

Abbreviations: *OS* Overall survival, *RFS* Relapse-free survival, *HR* Hazard ratio, *95% CI* 95% confidence interval, *nIL4Rα* Nuclear expression of IL4Rα, cIL4Rα; *nIL13Rα1* Nuclear expression of IL13Rα1

### Co-expression patterns of nuclear IL4Rα and nuclear IL13Rα1 are predictive for survival of soft-tissue sarcoma patients

**Fig. 3:**
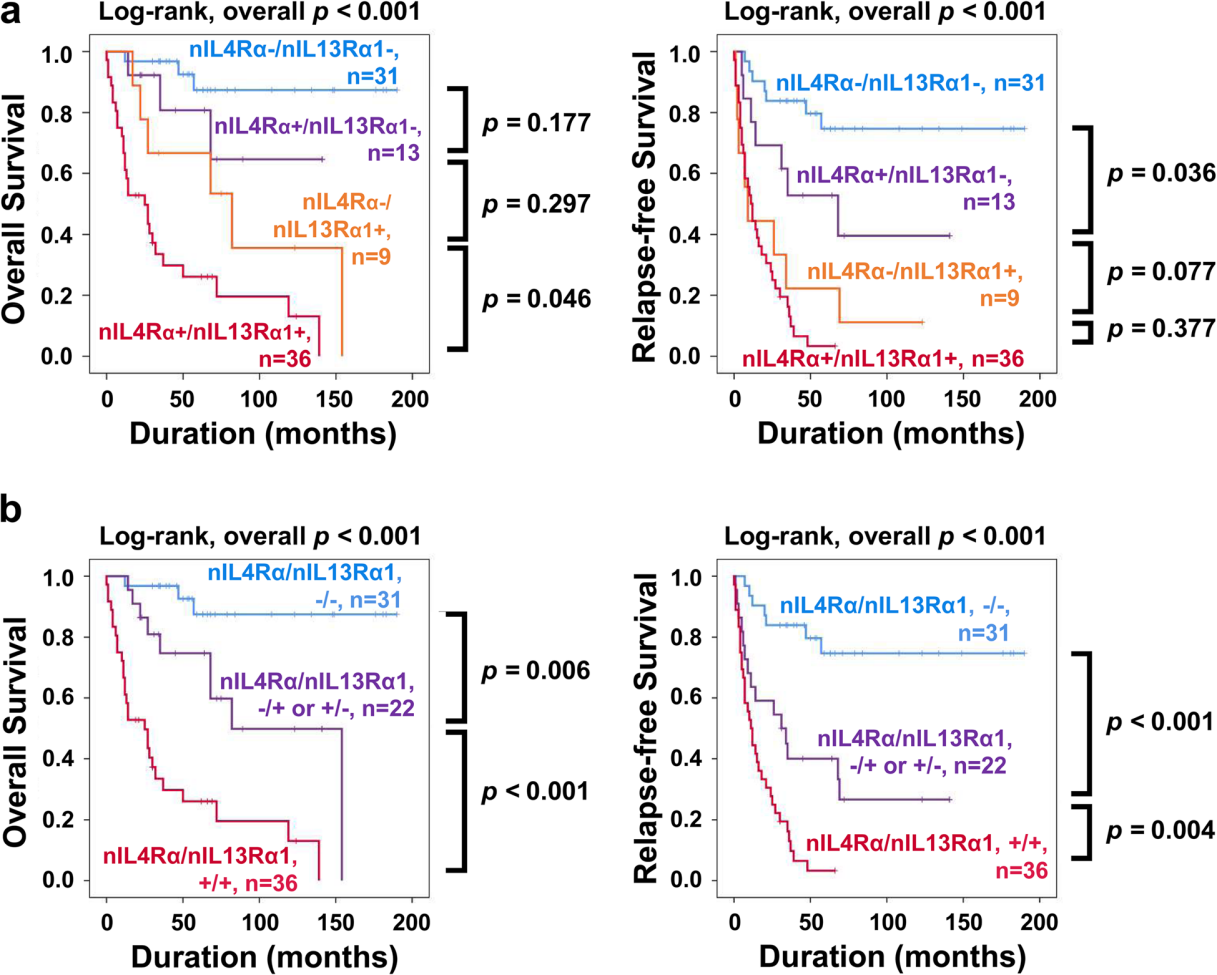
Survival analysis of overall survival and relapse-free survival of soft-tissue sarcomas according to the co-expression patterns of nuclear IL4Rα and nuclear IL13Rα1. **a** Kaplan-Meier survival curves for four subgroups of soft-tissue sarcomas according to the expression of nuclear IL4Rα (nIL4Rα) and nuclear IL13Rα1 (nIL13Rα1): nIL4Rα^−^/ nIL13Rα1^−^, nIL4Rα^−^/ nIL13Rα1^+^, nIL4Rα^+^/ nIL13Rα1^−^, and nIL4Rα^+^/ nIL13Rα1^+^ subgroups. **b** Kaplan-Meier survival curves in three subgroups of soft-tissue sarcomas: [nIL4Rα^−^/ nIL13Rα1^−^], [nIL4Rα^−^/ nIL13Rα1^+^ or nIL4Rα^+^/ nIL13Rα1^−^], and [nIL4Rα^+^/ nIL13Rα1^+^] subgroups

In multivariate analysis, the expression of nIL4Rα and nIL13Rα1 were the independent indicators of OS and RFS of STS patients. In addition, based on the molecular relationship between IL4Rα and IL13Rα1 as components of the type II IL4R complex and their possible roles in cancer progression [2–4], we evaluated the prognostic significance of the co-expression pattern of nIL4Rα and nIL13Rα1 in STSs. At first, we sub-classified STSs according to the co-expression patterns of nIL4Rα and nIL13Rα1 into four subgroups: nIL4Rα^−^/nIL13Rα1^−^, nIL4Rα^+^/nIL13Rα1^−^, nIL4Rα^−^/nIL13Rα1^+^, and nIL4Rα^+^/nIL13Rα1^+^. The nIL4Rα^−^/nIL13Rα1^−^ subgroup had the longest OS and RFS (10y-OS; 87%, 10y-RFS; 75%) and the nIL4Rα^+^/nIL13Rα1^+^ subgroup had the shortest OS and RFS (10-y-OS; 13%, 10y-RFS; 0%) (Table 5) (Fig. 3a). This subgrouping of STS was significantly associated with OS and RFS by both univariate and multivariate analysis (Multivariate analysis model 1: OS; overall *p <* 0.001, RFS; overall *p <* 0.001) (Table 6) (Fig. 3a). However, there was no significant difference in OS and RFS between the nIL4Rα^+^/nIL13Rα1^−^ subgroup and nIL4Rα^−^/nIL13Rα1^+^ subgroup (Fig. 3a). Therefore, based on these results, we re-grouped STSs into three prognostic sub-groups: (nIL4Rα^−^/nIL13Rα1^−^), (nIL4Rα^+^/nIL13Rα1^−^ and nIL4Rα^−^/nIL13Rα1^+^), and (nIL4Rα^+^/nIL13Rα1^+^). This subgrouping of STSs according to the co-expression patterns of nIL4Rα and nIL13Rα1 into three subgroups was significantly associated with OS and RFS by both univariate and multivariate analysis (Multivariate analysis model 2: OS; overall *p <* 0.001, RFS; overall *p* < 0.001) (Table 6) (Fig. 3b).

**Table 5:** Five- and ten-year overall survival and relapse-free survival according to co-expression patterns of nuclear IL4Rα and nuclear IL13Rα1

Co-expression pattern of nIL4Rα and nIL13Rα1	No.	5y-OS (%)	10y-OS (%)	5y-RFS (%)	10y-RFS (%)
Co-expression Model 1					
nIL4Rα/nIL13Rα1, −/−	31	87	87	75	75
nIL4Rα/nIL13Rα1,+/−	13	81	65	52	39
nIL4Rα/nIL13Rα1, −/+	9	67	36	22	11
nIL4Rα/nIL13Rα1, +/+	36	26	13	3	0
Co-expression Model 2					
nIL4Rα/nIL13Rα1, −/−	31	87	87	75	75
nIL4Rα/nIL13Rα1, +/− or −/+	22	75	50	40	27
nIL4Rα/nIL13Rα1, +/+	36	26	13	3	0

Abbreviations: *5y-OS* Overall survival rate at 5 years, *10y-OS* Overall survival rate at 10 years, *5y-RFS* Relapse-free survival rate at 5 years, *10y-RFS* Relapse-free survival rate at 10 years, *nIL4Rα* Nuclear expression of IL4Rα, *nIL13Rα1* Nuclear expression of IL13Rα1

**Table 6:** Univariate and multivariate Cox regression analysis of overall survival and relapse-free survival according to the co-expression patterns of nuclear IL4Rα and nuclear IL13Rα1 in soft-tissue sarcomas

Characteristics	No.	OS		RFS	
		HR (95% CI)	*p*	HR (95% CI)	*p*
Univariate analysis					
nIL4Rα/nIL13Rα1, −/−	31/89	1	< 0.001	1	< 0.001
+/−	13/89	3.230 (0.640–16.303)	0.156	2.990 (1.048–8.535)	0.041
−/+	9/89	7.673 (1.916–30.738)	0.004	7.670 (2.770–21.237)	< 0.001
+/+	36/89	18.871 (5.553–64.130)	< 0.001	10.815 (4.648–25.169)	< 0.001
nIL4Rα/nIL13Rα1, −/−	31/89	1	< 0.001	1	< 0.001
+/− or −/+	22/89	5.355 (1.434–20.004)	0.013	4.424 (1.801–10.871)	0.001
+/+	36/89	19.504 (5.671–67.080)	< 0.001	10.791 (4.633–25.134)	< 0.001
Multivariate analysis Model 1					
Distant metastasis, presence (vs. absence)		3.743 (1.762–7.952)	< 0.001	2.083 (1.127–3.849)	0.019
nIL4Rα/nIL13Rα1, −/−		1	< 0.001	1	< 0.001
+/−		3.140 (0.626–15.760)	0.165	2.972 (1.041–8.486)	0.042
−/+		2.808 (0.610–12.924)	0.185	4.827 (1.601–14.551)	0.005
+/+		11.927 (3.359–42.353)	< 0.001	8.729 (3.628–21.003)	< 0.001
Multivariate analysis Model 2					
Distant metastasis, presence (vs. absence)		3.663 (1.836–7.307)	< 0.001	2.312 (1.305–4.095)	0.004
nIL4Rα/nIL13Rα1, −/−		1	< 0.001	1	< 0.001
+/− or −/+		2.947 (0.750–11.584)	0.122	3.619 (1.447–9.052)	0.006
+/+		12.004 (3.396–42.433)	< 0.001	8.397 (3.491–20.197)	< 0.001

Abbreviations: *OS* Overall survival, *RFS* Relapse-free survival, *HR* Hazard ratio, *95% CI* 95% confidence interval, *nIL4Rα* Nuclear expression of IL4Rα, cIL4Rα; *nIL13Rα1* Nuclear expression of IL13Rα1

## Discussion

In this study, we have shown that the expression of IL4Rα and IL13Rα1 are associated with clinicopathological factors related to the progression of STSs, and there was a significant association between the expression of IL4Rα and IL13Rα1 in STSs. Furthermore, there was a positive correlation between the expression of mRNA IL4Rα and IL13Rα1 in glioblastoma multiform [9]. In addition, the expression of IL4Rα and IL13Rα1 were increased in meningioma compared with normal brain tissue [8] and were higher in invasive pituitary adenoma compared to non-invasive pituitary adenoma [7]. In STSs, the expression of mRNA of IL4Rα and IL13Rα1 were higher compared with normal counterpart tissue [STS versus normal (TPM, median expression): IL4Rα; 31.19 for STS and 23.15 for normal, IL13Rα1; 31.15 for STS and 17.33 for normal] and there was a significant correlation between the expression of IL4Rα and IL13Rα1 (Pearson’s *R* = 0.15, *p* = 0.016) in the GEPIA public database (http://gepia.cancer-pku.cn. accessed November 15, 2020) [26]. In addition, higher expression of IL4Rα and IL13Rα1 were associated with advanced clinicopathological factors of STSs such as higher tumor stage, cancer metastasis, higher histologic grade, increased mitosis, and tumor necrosis. Furthermore, nuclear and cytoplasmic expression of IL4Rα and IL13Rα1 were associated with shorter survival of STSs. Especially, individual and combined expression patterns of nuclear IL4Rα and IL13Rα1 were independent indicators of poor prognosis of STS patients. Consistently, although nuclear and cytoplasmic expression were not analyzed separately, higher expression of IL4Rα and IL13Rα1 were significantly associated with shorter cancer-specific survival and RFS of clear cell renal cell carcinoma patients [12]. Especially, clear cell renal cell carcinoma patients with co-positivity for the expression of IL4Rα and IL13Rα1 had the shortest survival time [12]. In addition, the prognostic significance of individual expression of IL4Rα or IL13Rα1 has been reported in various human cancers. Higher expression of mRNA and protein of IL4Rα was associated with shorter survival of mesothelioma patients [10]. In breast cancer, higher expression of IL13Rα1 was significantly associated with shorter OS and disease-specific survival [11]. Higher expression of IL13Rα1 mRNA was associated with poor prognosis of glioblastoma patients [9]. Therefore, targeting the IL4R complex might be a therapeutic strategy for cancers with poor prognosis that highly express IL4Rα and IL13Rα1.

The prognostic impact of the expression of IL4Rα and IL13Rα1 in human cancers is related to the role of IL4Rα/IL13Rα1 in cancer-related signaling. Although studies on the role of IL4Rα/IL13Rα1 in STSs has been limited, it has been reported that the IL4Rα/IL13Rα1 receptor complex is involved in tumorigenesis via mechanism the cell cycle, apoptosis, and cellular proliferation [1, 2, 12]. In renal cell carcinoma cells, knock-down of IL4Rα or IL13Rα1 induced cell cycle arrest and apoptosis by suppressing JAK2-mediated phosphorylation of FOXO3 [2]. In rhabdomyosarcoma cells, activation of IL4R with IL4 and IL13 ligands increased tumor growth through activation of STAT6, Akt, or MAPK pathways [16]. In 4 T1 breast cancer cells, IL4Rα enhanced tumor growth by mediating IL4-related enhancement of glucose and glutamine metabolism [27]. The silencing of IL4Rα inhibited the growth and invasiveness of pancreatic cancer cells by suppressing the STAT3 and Akt pathways [28]. In colorectal cancer cells, IL13 induced epithelial-to-mesenchymal transition through the STAT6 pathway and was reversed with knock-down of IL13Rα1 [29]. However, there are controversial reports on the role of IL4R in tumorigenesis. In a transgenic mouse model with overexpression of IL4, IL4/IL4Rα suppressed the development of melanoma through activation of the P21-mediated STAT6 pathway and inhibition of anti-apoptotic BCL2 expression [30]. In addition, reduction of IL4R signaling was associated with increased initiation of colorectal cancer development, but reduced cancer progression [31]. This report emphasized that a therapeutic approach carefully targeting IL4R signaling according to the cancer progression stage could be effective [31]. Therefore, although most reports suggest the IL4R complex as a promising therapeutic target of human cancers, a tailored approach according to the specific subtype of cancer is likely to be the most effective.

In our results, both nuclear and cytoplasmic expression of IL4Rα and IL13Rα1 were significantly associated with the survival of STS patients. When considering the role of type II IL4R as a receptor for cytokines, IL4Rα and IL13Rα1 are expected to be localized in the cytoplasmic membrane. However, in this study, their expression in nuclei presented as a more powerful prognostic indicator of STSs compared with their cytoplasmic expression. Therefore, when we searched for the subcellular localization of IL4Rα and IL13Rα1 in a public database, nuclear expression of IL4Rα and IL13Rα1 was presented in The Human Protein Atlas database (https://www.proteinatlas.org. accessed November 15, 2020) [5, 32]. In addition, the expression of IL4Rα and/or IL13Rα1 was observed in both the cytoplasm and nuclei of human cancer tissue samples, such as clear cell renal cell carcinoma [12], squamous cell carcinoma [13], and lung cancer [33]. Moreover, when considering the nuclear and cytoplasmic expression of the molecules related to IL4Rα/IL13Rα1 such as JAK2 and STAT6 based on The Human Protein Atlas, the expression of IL4Rα/IL13Rα1 was expected in both cytoplasm and nuclei [5, 32]. Therefore, it is suggested that the nuclear localization of IL4Rα and IL13Rα1 might have a role in the progression of cancers. However, the significance of the nuclear localization of IL4Rα and IL13Rα1 in the progression of cancer is not clear. One possible explanation might be that IL4Rα/IL13Rα1 are involved in tumorigenesis in association with nuclear proteins related to tumor biology [12]. Recently, it has been reported that IL4Rα/IL13Rα1interact with nuclear protein JAK2 and FOXO3 [33]. In renal cell carcinoma cells, the silencing of IL4Rα expression reduced interaction between JAK2 and FOXO3 and resulted in stabilizing FOXO3 [7]. Therefore, when considering the oncogenic role of JAK2 and tumor-suppressive role of FOXO3, nuclear localization of IL4Rα/IL13Rα1 exerts its role by involving JAK2-FOXO3 interaction in the progression of STSs. However, despite the presence of cytoplasmic and nuclear expression of IL4Rα and IL13Rα1 in human cancers, there have been no reports specifically focused on the effects of subcellular localization of IL4Rα and IL13Rα1 in human cancers. Therefore, further study is needed to clarify the role of cytoplasmic and nuclear expression of IL4Rα and IL13Rα1 in human cancers.

In this study, higher expression of IL4Rα and IL13Rα1 were associated with progression and poor survival of STS patients. Therefore, IL4Rα/IL13Rα1 might be a potential therapeutic target for STS patients. Based on the characteristics of the IL4Rα/IL13Rα1 receptor complex that is activated by both IL4 and IL13 and it stimulates the JAK1/JAK2/STAT6 pathway in solid cancers, IL4/IL13, IL4Rα/IL13Rα1, and JAK1/JAK2/STAT6 might be good therapeutic targets for the treatment of malignant tumors expressing IL4Rα/IL13Rα1. In rhabdomyosarcoma cells, IL4 and IL13 activate cellular proliferation through the JAK/STAT signaling pathway, and blocking IL4R with a neutralizing antibody suppressed tumor progression [16]. Blocking of IL4Rα also induced the apoptosis of breast cancer cells [34, 35]. In renal cell carcinoma cells, knock-down of IL4Rα or IL13Rα1 and pharmacological inhibition of JAK2 induced cell cycle arrest and apoptosis of cancer cells [12]. Similarly, inhibition of JAK2, which is downstream of IL4R, delayed tumor growth in an osteosarcoma xenograft model [17]. In addition, as IL4R is highly expressed in human cancers, receptor-directed anti-tumor therapeutic approaches have been tested. AP-1 (human atherosclerotic plaque-specific peptide-1)-conjugated liposomal conjugate specifically targeted at IL4Rα, showed an anti-cancer effect on IL4Rα-overexpressing colon cancer cells [36]. Furthermore, with respect to treatment of human cancers, one of the important aspects for archiving successful treatment is overcoming the resistance of cancer cells to anti-cancer therapeutics; thus, the regulation of host anti-immune mechanisms is one of the promising therapeutic strategies, and IL4R also might be a potential target to overcome cancer resistance [3]. Colorectal cancer-related cancer-initiating cells evade immune surveillance through IL4/IL4R-mediated inhibition of T cell proliferation [37]. Blocking of IL4R with IL4Rα antagonist or anti-IL4 neutralizing antibodies sensitized CD133-expressing colon cancer stem cells to conventional the chemotherapeutics oxaliplatin and 5-FU [38]. Therefore, when considering the shorter survival of STS patients expressing IL4Rα and IL13Rα1, therapeutics targeting IL4Rα and IL13Rα1 might be novel therapeutic strategems for the treatment of STSs. However, despite the prognostic impact of the expression of IL4Rα and IL13Rα1 in STSs, our study has a limitation: the cases in this study are heterogeneous. When considering profound biological differences among different STS types, further study is needed to investigate the expression and role of IL4Rα and IL13Rα1 in specific types of STSs.

## Conclusions

In conclusion, this study demonstrated that the expression of IL4Rα and IL13Rα1, especially when highly expressed in nuclei, were associated with advanced clinicopathological factors of STS such as higher tumor stage and high histologic grade, and predicted shorter survival of STS patients. Therefore, the expression of IL4Rα and IL13Rα1 might be used as novel prognostic indicators for STS patients. In addition, this study suggests that blocking of the IL4Rα/IL13Rα1 pathway might be a novel therapeutic stratagem for STSs.

## Data Availability

The datasets generated during and/or analyzed during the current study are available from the corresponding author on reasonable request.

## Abbreviations

95% CI: 95% confidence interval
cIL13Rα1: Cytoplasmic expression of IL13Rα1
cIL4Rα: Cytoplasmic expression of IL4Rα
HR: Hazard ratio
IL13: Interleukin-13
IL13R: Interleukin-13 receptor
IL4: Interleukin-4
IL4R: Interleukin-4 receptor
nIL13Rα1: Nuclear expression of IL13Rα1
nIL4Rα: Nuclear expression of IL4Rα
OS: Overall survival
RFS: Relapse-free survival
ROC: Receiver operating characteristic
STS: Soft-tissue sarcoma
